# Postoperative complications following surgery for congenital hyperinsulinism and insulinomas in pediatric patients

**DOI:** 10.1007/s00383-025-06191-8

**Published:** 2025-09-17

**Authors:** Gregers Gaardskær Boel, Henrik Thybo Christesen, Mark Bremholm Ellebæk, Michael Bau Mortensen

**Affiliations:** 1 Medical Student, Odense University Hospital, 5000 Odense, Denmark; 2 Odense Pancreas Center (OPAC), Department of Clinical Research, Faculty of Health Sciences, Odense University Hospital, University of Southern Denmark, Odense, Denmark; 3 Hans Christian Andersen Children’s Hospital, Odense University Hospital, Odense, Denmark; 4 Research Unit for Surgery, Odense University Hospital, University of Southern Denmark, and Centre of Excellence in Gastrointestinal Diseases and Malformations in Infancy and Childhood (GAIN), Odense University Hospital, University of Southern Denmark, Odense, Denmark; 5 Research Unit for Surgery, Odense PIPAC Center (OPC) and Odense Pancreas Center (OPAC), Odense University Hospital, University of Southern Denmark, Odense University Hospital, Odense, Denmark; 6 Institute of Clinical Research, Faculty of Health Sciences, Denmark and Department of Surgery, HPB and Upper GI Section, University of Southern, Odense University Hospital, Odense, Denmark

**Keywords:** Congenital Hyperinsulinism, Near-total pancreatectomy, Postoperative complications, Pancreatic surgery, Pediatric surgery

## Abstract

**Purpose:**

To systematically describe postoperative complications in surgery for HI in pediatric patients.

**Methods:**

In this retrospective single-center study, we systematically analyzed the rate and grade of complications according to the Comprehensive Complication Index (CCI), Clavien-Dindo Classification (CDC), and the Clavien-Madadi Classification (CMC) in 74 patients undergoing a total of 89 surgeries for CHI (*N* = 68) or pediatric insulinomas (*N* = 6) at Odense University Hospital, Denmark, from 01.01.2010 until 01.10.2024.

**Results:**

Patients surgically treated for focal CHI had more favorable surgical outcomes with a mean CCI score of 10.8 vs. the diffuse CHI mean CCI of 26.3 (*p* = 0.0018). Surgical treatment for pediatric insulinomas resulted in a mean CCI of 28.9. In the total group, the most common complication was postoperative infection (29%), followed by delayed gastric emptying (20%). The rate of postoperative pancreatic fistula was 11%, but only 3.4% were clinically relevant. Eight percent of the surgical procedures resulted in complications classified as CMD grade IIIb or higher.

**Conclusion:**

Despite the complex nature of surgery in pediatric patients with CHI or insulinomas, the majority had an uneventful recovery. Severe complications (CMC grade IIIb +) were, however, seen in 8%. Prospective, systematic postoperative complication score evaluation is encouraged in surgery for pediatric HI.

## Introduction

Despite being a rare condition affecting only 1 in 35.000 births, congenital hyperinsulinism (CHI) is the most common cause of persistent hypoglycemia in infants and children, with a high risk of seizures and neurological impairment if not promptly recognized and treated [1, 2].

The incidence of insulinomas is estimated to be four per million/year with only 6% of cases occurring in children and adolescents under 20 years of age [3]. Data on pediatric insulinomas is generally lacking with only a few case reports and cohorts described [4]. Given the rarity and complexity, surgery in pediatric patients for these rare conditions should be centralized to experienced centers. The indication for and extend of surgery varies for the subtype of CHI. Diffuse CHI affects all β-cells in the pancreas, whereas focal CHI usually presents with a very small focal insulin-producing lesion. In atypical (histologically non-diffuse, non-focal) CHI, varying degrees of endocrine cells can be affected, at times with macroscopic overgrowth. In general, CHI patients unresponsive to the first drug-of-choice diazoxide are potentially candidates for pancreatic surgery [1]. In these patients, a thorough diagnostic work-up is needed to distinguish between the different subtypes.

The subtyping includes genetic analysis, as a paternal mutation in K_ATP_-channel genes *ABCC8* or *KCNJ11* predicts focal CHI, in which a somatic second hit with a focal loss of the maternal allele on chromosome 11p15 causes loss of heterozygosity for the K_ATP_-channel gene mutation. To further identify the presence and location of a focal lesion, 18F-DOPA PET-CT is used prior to surgery in specialized centers with a sensitivity of 75% to 100% and a specificity of 88–100% for the detection of the focal lesion [5]. For these patients, resection *in toto* of the focal pancreatic lesion is curative.

In patients with diffuse CHI, pancreatic surgery is less attractive as 95–98% of the pancreas is typically removed in a near-total pancreatectomy, which in many cases results in postoperative normoglycemia, but with a well-known long-term risk for diabetes and malabsorption [6]. In diffuse CHI patients unresponsive to the often complex medical and dietary treatment, or with limited access to optimal conservative treatment [7], surgery is still indicated [1].

In pediatric patients with insulinoma, surgery is the treatment of choice and curative as for focal CHI [8], however, with a risk relapse in a minority of pediatric insulinoma patients with multiple endocrine neoplasia type 1.

Surgery of the pancreas is complex and requires special attention in terms of postoperative complications in both children and adults. While complications have been intensively studied and classified in the latter group, no detailed evaluation, consensus classification or grading of complications following pancreatic surgery has been determined or suggested in infants, children and adolescents. This is particularly relevant for patients who have undergone surgical intervention for CHI and insulinomas, where existing evidence is scarce and lacks standardized outcome grading.

To optimally monitor these patients’ postoperative course and surgical outcome, a thorough mapping and grading of the most common and most severe complications in the postoperative period is needed. We therefore aimed to use strict pre-defined complication definitions and grading systems to evaluate the postoperative complications after surgical treatment for CHI and pediatric insulinomas in our expert center.

## Methods

### Study design and setting

This is a retrospective single-center study from patients with HI hospitalized at Odense University Hospital, Denmark, since 2024 designated as one of only eight Centers of Excellence for treatment and research in CHI in the world by the patient organization Congenital Hyperinsulinism International [9]. Data for the study were retrieved from patients’ medical journals in the Danish national electronic medical records, covering the period from January 01, 2010 to October 01, 2024. The study was reported according to The Strengthening the Reporting of Observational Studies in Epidemiology (STROBE guidelines) for cohort studies [10].

### Surgical procedures and multi-disciplinary team

Surgery was conducted via a supraumbilical transverse incision followed by entry into the lesser sac exposing both upper and lower borders of the gland. Together with an extensive Kocher’s maneuver complete visual and tactile evaluation of the gland was possible. In cases where diagnostic work-up with PET-CT and genetics predicts focal CHI or insulinomas, intraoperative ultrasound was used to localize the lesion [11] and to plan resection or enucleation based on local anatomy, including distance to the main pancreatic duct and the bile duct. The suspected lesion was removed along with lymph nodes in close proximity and sent for frozen-section analysis. The surgery was completed when frozen-section analysis confirmed the diagnosis and proof of radical resection, via free resection borders. Major local enucleation cavities were closed using 5–0 or 6–0 single or running monofilament sutures. More extensive resections (e.g. sub-total pancreatectomy, pancreaticoduodenectomy, distal pancreatectomy) and variations thereof were performed according to standard procedures and drains were placed at the discretion of the surgeons involved. In cases with intra-operative confirmation of diffuse CHI, a near-total pancreatectomy was performed with removal of up to 98% of the pancreatic tissue. The preoperative multi-disciplinary assessment decided the intended extent of resection on a case-to-case basis aiming for either postoperative normoglycemia or a sufficient response to pharmacological treatment. The surgical approaches were divided into three groups based on the extend and complexity of the resection: 1) Enucleations 2) Medium extensive surgery (i.e. distal pancreatectomy, subtotal pancreatectomy: < 90%) and 3) Extensive surgery (i.e. near-total pancreatectomy: > 90%, Whipple’s procedure, duodenum preserving head resection).

Treatment for CHI and insulinomas is highly specialized requiring a multidisciplinary approach, involving pediatric and pancreatic surgical teams, pediatric endocrinology, clinical genetics, pathology, nuclear medicine, radiology and intensive care. In addition, the rarity of CHI and insulinomas necessitates a concentration of cases at a dedicated facility. The International Hyperinsulinism Center at Odense University Hospital (OUH) is the tertiary referral center for the treatment of hyperinsulinism in Denmark and is recognized as a center of excellence by Congenital Hyperinsulinism International[12]. The center receives and treats patients referred from countries throughout Europe.

### Patients

Inclusion criteria: All patients below 18 years of age referred to the center and undergoing surgery for HI were included in the study.

Exclusion criteria: Non-pancreatic surgery, non-insulinoma persistent hyperinsulinemic hypoglycemia syndrome (ni-PHHS) by histological examination.

Patient data were obtained from national electronic medical records. A 30 days follow-up period was set with the date of surgery being defined as the index date. Subsequent pancreatic surgeries were treated as separate events with separate complication registration. Patients discharged prior to day 30 were registered as having no additional complications unless additional communication between their primary hospital and our center was noted. Data collection was done through a dedicated REDCap data capture tool hosted by the Open Patient Data Explorative Network (OPEN) [13, 14]. Data collection and grading of complications were done by the main author with cases of doubt being decided collectively between all authors.

### Definitions and classifications

Postoperative complications are defined as any deviation from the ideal (expected) postoperative surgical course. This does not include failure to cure or recurrent disease, since these findings are not related to the surgical procedure per se. Based on well-known, defined, and rated complications to pancreatic surgery in adults, we chose to include the following complications as defined by the International Study Group of Pancreatic Surgery (ISGPS).

1. **Postoperative Pancreatic Fistula** (POPF): Any measurable volume of fluid on or after postoperative day 3 with an amylase content greater than 3 times the concentration of serum amylase, Graded A-C. Grade A being biochemical changes only. Grade B requires a change in management or adjustment of the clinical pathway and grade C a major change in the clinical pathway and clinical stability is borderline [15].
2. **Chyle leak:** Output of milky-colored fluid from a drain, drain site, or wound on or after postoperative day 3, with a triglyceride content ≥ 110 mg/dL (≥ 1.2 mmol/L). Graded A-C. Grade A has no specific intervention other than oral dietary restrictions. Grade B being a prolongation of the hospital stay or naso-enteral nutrition with dietary restriction, total parenteral nutrition, octreotide, maintenance of surgical drains, or placement of new percutaneous drains. Grade C is reserved for more invasive in-hospital treatment, ICU admission, or mortality [16].
3. **Bile leak:** Bilirubin concentration in the drain fluid at least 3 times the serum bilirubin concentration on postoperative day 3 or as the need for radiologic or operative intervention resulting from biliary collections or bile peritonitis. Graded A-C. Grade A is defined as no change in the clinical course. Grade B requires active therapeutic intervention excluding re-laparotomy, whereas grade C requires re-laparotomy [17].
4. **Postpancreatectomy hemorrhage:** Defined by three parameters: onset, location, and severity. The onset is either early (≤ 24 h within index operation) or late (> 24 h). The location is either intraluminal (e.g. hemosuccus pancreaticus) or extraluminal. The severity of the bleeding is graded A-C. With Grade A having no therapeutic consequence. Grade B requires blood transfusion intermediate care or ICU treatment. Grade C requires embolization, endoscopy or re-laparotomy [18].
5. **Delayed Gastric Emptying** (DGE): Inability to return to a standard diet by the end of the first postoperative week and includes prolonged nasogastric intubation of the patient. Graded A-C. Grade A being the inability to return to standard diet by postoperative day (POD) 7, grade B by POD 14 and grade C by POD 21. The complication will be defined as the onset of DGE after surgery [19].
6. **Postpancreatectomy Acute Pancreatitis** (PPAP): Sustained postoperative serum hyperamylasemia greater than the institutional upper limit of normal for at least the first 48 h postoperatively, associated with clinically relevant features and radiologic alterations consistent with PPAP. Graded A-C, with grade A being biochemical changes only. Grade B is mild or moderate complications and grade C is defined as severe life-threatening complications that lead to persistent organ faliure [20].

In addition, complications were graded in severity according to the Clavien-Dindo classification (CDC) [21] and the Clavien-Madadi classification (CMC) [22], the latter is specifically designed for postoperative complications in pediatric surgery. The main difference is complication Grade IIIa or IIIb, with CMC differing from CDC by open versus closed interventions, instead of the type of anesthesia. Furthermore, complications were divided into two groups according to the severity (*I-IIIa* and *IIIb and above*) for both the CDC and CMC.

A comprehensive complication index (CCI) was calculated using a specific formula, which sums a single patient’s CDC complications giving a continuous scale between 0 and 100; consequently, the CCI gives an estimate for the cumulative burden of all postoperative complications encountered in a single case[23]. The CCI is not yet commonly used in pediatric pancreatic surgery. However, it has in adult populations, been proven to be more accurate for evaluating the risk of a prolonged length of stay than the use of the CDC alone [24].

### Statistical analysis

The incidence of each separate complication was recorded, and the rates were calculated as a percentage. All surgeries were pooled to achieve a total complication burden. The mean CCI of each group is calculated using the AssesSurgery CCI calculator [25]. Microsoft Excel was used to perform a two-sided homoscedastic t-test comparing the CCI of diffuse CHI and focal CHI.

### Ethical aspects

The study was approved by the Regional Data Protection Agency (project ID: 24/16225) and the Regional Committees on Health Research Ethics for Southern Denmark (project ID: 24/10264) and performed in conformity with the Declaration of Helsinki’s description of ethical principles. The IT department at Odense University Hospital identified and provided the patients based on ICD-10 and relevant procedure codes.

## Results

**Fig. 1:**
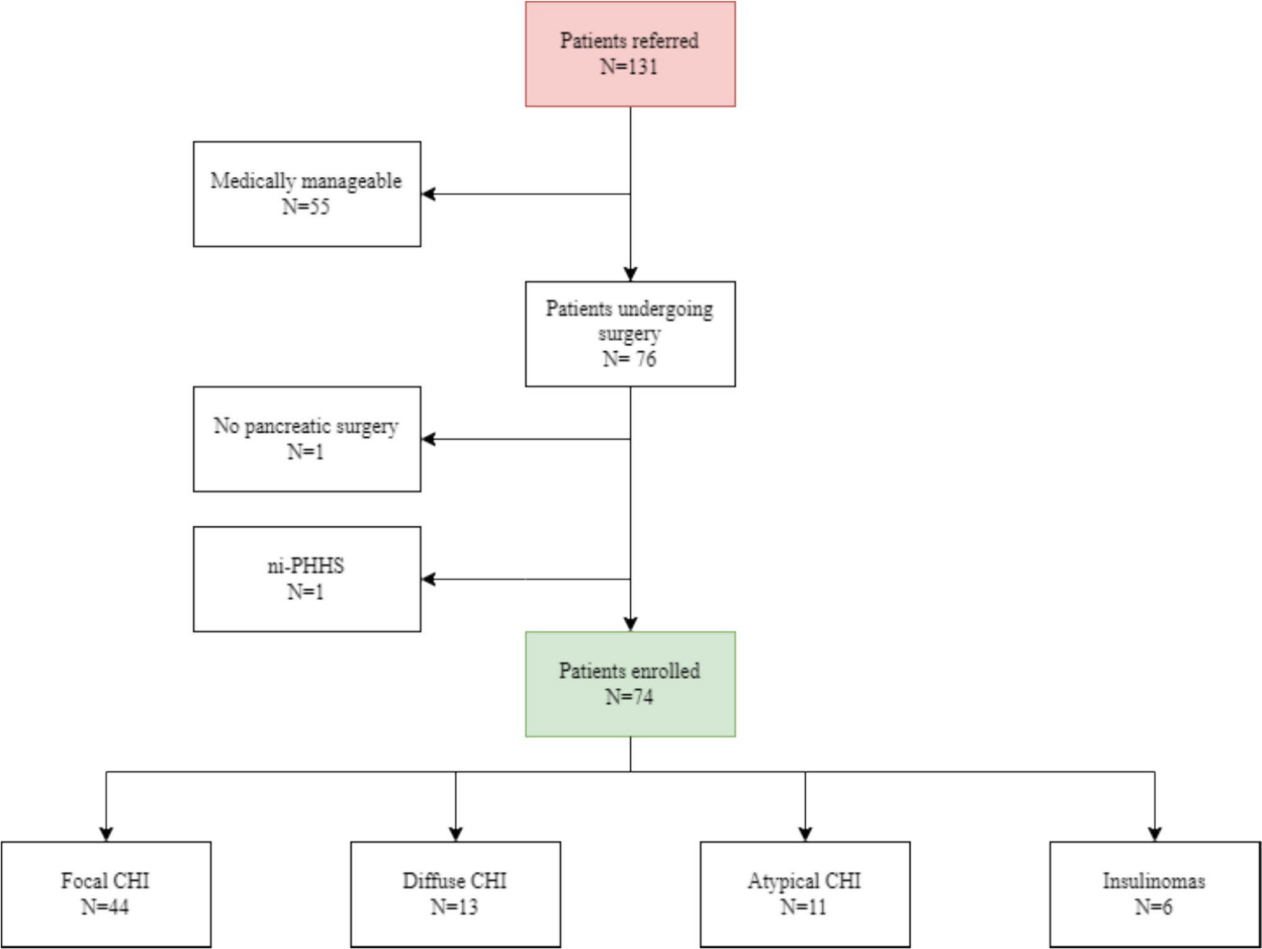
Patient inclusion Ni-PHHS; non-insulinoma pancreatic hyperinsulinemic hypoglycemia syndrome. Atypical CHI defined as non-focal, non-diffuse CHI

Between January 2010 and October 2024, a total of 131 patients were referred to the International Hyperinsulinism Center at OUH with various forms of hyperinsulinism. Fifty-five of the patients were medically manageable and excluded from the study. A single patient underwent non-pancreatic surgery and was excluded. A patient undergoing surgery for presumed insulinoma, which was later diagnosed with non-insulinoma persistent hyperinsulinemic hypoglycemia syndrome (ni-PHHS) and was also excluded. The remaining 74 patients (44 focal CHI, 13 diffuse CHI, 11 atypical CHI and 6 insulinomas) were included in the study (Fig. 1). Patient characteristics are listed in Table 1.

**Table 1:** Patient Characteristics

	Focal CHI	Diffuse CHI	Atypical CHI	Insulinoma	Total
Number of patients (%)	44 (59.5)	13 (17.6)	11 (12.4)	6 (6.7)	74 (100)
Female gender N (%)	25 (54.5)	6 (46.2)	9 (81.8)	4 (75.0)	44 (58.7)
Birthweight (g) Mean (range)	3505 (2180–4650)	4060 (2638–5200)	3340 (2185–4800)	N/A	3579 (2180–5200)
Paternal KATP mutation^1^ N (%)	43 (97.7)	2 (15.4)	4 (36.4)	0 (0)	49 (65.3)
Age at surgery (Months) Median (Range)	5.2 (0.8–28.8)	5.7 (1.2–35.8)	10 (0.7–26.6)	184.4 (173–200.3)	6.3 (0.7–200.3)
Length of stay (Days) Mean (range)	15 (4–82)	26 (9–70)	13 (8–30)	8 (4–14)	16 (4–91)

^a^Verified paternal mutation in the *ABCC8* or *KCNJ11* gene.

## Surgical treatment

### Focal CHI

Forty-four patients were surgically treated for focal CHI, with 38 (86%) undergoing a focal enucleation of the presumed lesion. Thirty-six of the patients were completely cured by the first surgery with 8 (18%) requiring additional resection of pancreatic tissue and a single patient requiring a total of three surgeries. All 44 patients with focal CHI were completely cured of their hyperinsulinism without the need for further medical treatment.

### Diffuse CHI

Thirteen patients were treated for diffuse CHI. The surgical approach varied from case to case, and the assessed removal of pancreatic tissue varied between 50 and 100%. Due to persistent hypoglycemia, one patient required two resections and another patient three resections. After the final surgery, a total of 4 (29%) of the diffuse CHIs became euglycemic at 30-day follow-up, with 8 (57%) having medically manageable hyperinsulinemia and 2 (14%) patients having insulin-dependent hyperglycemia, one of which relapsed to hypoglycemia at 3 months post-surgery.

### Atypical CHI

Eleven patients were treated for atypical CHI. Four of the cases were recognizable as atypical CHI prior to surgery. The remaining cases presented by 18F-DOPA PET/CT and by genetic predictions as either focal or diffuse. Six patients received distal pancreatectomies as the pancreatic tail was suspected hyperinsulinemic, two patients were treated with a localized resection akin to treatment for focal CHI and three patients were treated with near total pancreatectomies. A single case treated with a distal pancreatectomy required an additional operation and further resection of the pancreatic tail. At 30-day follow-up from last surgery 6 (55%) were cured and 5 (45%) having medically manageable hypoglycemia.

### Insulinomas

Six patients were surgically treated for insulinomas with one of the patients having both a glucagonoma and insulinoma due to a Multiple Endocrine Neoplasia (MEN1) mutation. Of the five patients without the MEN1 mutation, three were treated by simple enucleation of the insulinoma. The two other patients underwent, distal pancreatectomy and a Roux-en-y pancreatojejunostomy, both with complete cure. The MEN1 patient was presumed to be treated by enucleation of a focal lesion in the uncinate process. However, pathology revealed the removed lesion to be a glucagonoma. Two subsequent surgeries were performed ending with a complete Whipple’s procedure. Postoperative histology of the exercised tissue showed a “Horseshoe-like” lesion, possibly two connected focal lesions. All patients were completely cured of their hyperinsulinism at 30-day follow-up from their final surgery.

### Complications

In this cohort of 74 pediatric patients undergoing a total of 89 surgical procedures for CHI or insulinomas. The most commonly encountered complication was non-surgical infections (29.2%), including central line-associated bloodstream infections. Delayed gastric emptying was present after 20.2% of surgeries as the second most common complication. Overall, the risk of surgically induced abscesses was low (5.6%) (Table 2). Eleven percent of surgeries resulted in pancreatic fistulas according to the ISPGS definition. However, only 3.4% were above grade A, thus having an impact on the clinical course (Table 3). The most important severe complications encountered were intra-abdominal abscess (5.6%), sepsis (3.4%), POPF Grade B + C (3.4%).

**Table 2:** Postoperative complications ≤ 30 days after surgery

	First surgery	Second surgery	Third surgery	Total (%)
**Number of surgeries**	**74 (100)**	**12 (100)**	**3 (100)**	**89 (100)**
Infection with unknown focus	9 (12.2)	2 (16.7)	1 (33.3)	12 (13.4)
Central line associated bloodstream infection	10 (13.5)	1 (8.3)	0 (0)	11 (12.2)
Exocrine pancreas insufficiency	5 (6.8)	2 (16.7)	1 (33.3)	8 (9.0)
Wound infection	4 (5.4)	1 (8.3)	0 (0)	5 (5.6)
Intra-abdominal abscess	4 (5.4)	0 (0)	1 (33.3)	5 (5.6)
Small bowel obstruction	4 (5.4)	0 (0)	0 (0)	4 (4.5)
Sepsis	3 (4.1)	0 (0)	0 (0)	3 (3.4)
Other^a^	2 (2.7)	1 (8.3)	0 (0)	3 (3.4)
Anemia	2 (2.7)	1 (8.3)	0 (0)	3 (3.4)
Peritonitis	1 (1.4)	0 (0)	0 (0)	1 (1.1)
Endocarditis^2^	1 (1.4)	0 (0)	0 (0)	1 (1.1)
Endocrine pancreas insufficiency	0 (0)	0 (0)	1 (33.3)	1 (1.1)
ISGPS complications				
Delayed gastric emptying	14 (18.9)	3 (25)	1 (33.3)	18 (20.2)
Postoperative pancreatic fistula	6 (8.1)	3 (25)	1 (33.3)	10 (11.2)
Chyle leak	3 (4.1)	0 (0)	0 (0)	3 (3.4)
Post pancreatectomy acute pancreatitis	1 (1.4)	0 (0)	0 (0)	1(1.1)
Post pancreatectomy hemorrhage	0 (0)	0 (0)	0 (0)	0 (0)
Bile leak	0 (0)	0 (0)	0 (0)	0 (0)

Data presented as N (%)

Percentages are calculated as percent of surgeries which experience the complication

^a^One case of stridor and tachypnea treated with inhalation of adrenalin, one case of nausea and one larynx spasm with intubation

bThe patient had severe hypertrophic cardiomyopathy prior to surgery

**Table 3:** Classification of postoperative complications according to the International Study Group for Pancreatic Surgery (ISGPS)

	POPF^a^	Bile leak	Chyle leak	PPH^b^	DGE^c^	PPAP^d^
Grade A	7 (7.8)	0 (0)	0 (0)	0 (0)	10 (11.2)	0 (0)
Grade B	2 (2.2)	0 (0)	3 (3.4)	0 (0)	4 (4.5)	1 (1.1)
Grade C	1 (1.1)	0 (0)	0 (0)	0 (0)	4 (4.5)	0 (1.1)

Surgeries for focal CHI had the lowest severe complication rate of 3.8% (CMC) and 7.5% (CDC), along with a mean CCI of 10.82, while surgeries for diffuse CHI had a significant higher complication rate of 13.3% (CMC) and 13.3% (CDC), with a mean CCI of 26.25 $(p\approx \text{0,0018}$). More than half of the total surgeries resulted in postoperative complications within 30 days. However, the vast majority of complications were rated low in both CDC and CMC (grade I-IIIa), and only 8% and 12% of the complications required significant interventions according to CMC and CDC, respectively (Table 4).

**Table 4:** Grading of the single most severe complication

Number of surgeries	Diffuse *N* = 15	Focal *N* = 53	Atypical *N* = 13	Insulinoma *N* = 8	Total *N* = 89
Clavien-Madadi classification(worst)					
Overall complication rate I-V^a^	13 (86.6)	21 (39.6)	7 (53,8)	7 (87.5)	48 (54.0)
I-IIIa	11 (73.3)	18 (34.0)	6 (46.2)	6 (75)	41 (46.1)
≥ IIIb	2 (13.3)	2 (3.8)	1 (7.7)	1 (12.5)	6 (7.9)
Clavien-Dindo Classification(worst)					
Overall complication rate I-V	13 (86.6)	21 (39.6)	7 (53.8)	7 (87.5)	48 (54.0)
I-IIIa	11 (73.3)	16 (30.1)	6 (46.2)	4 (50)	37 (41.6)
≥ IIIb	2 (13.3)	4 (7.5)	1 (7.7)	3 (37.5)	10 (12.4)
Mean comprehensive complication index^2^	26.25	10.82	13.71	28.93	15.46

Data presented as N(%).

^a^The number of surgeries which resulted in at least one complication

^b^CCI comprised of all complications, not the single worst

Patients undergoing enucleations experienced few overall complications at 39.1% and very few severe complications at CDC and CMC > IIIb at 4.35%. The rate of severe complications was higher in the medium extensive group at 6.9% (CMC) and 13.8% (CDC) and highest in the extensive surgery group at 14.3% (CMC) and 28.6% (CDC) (Table 5).

**Table 5:** Grading of the single worst complication per surgery, divided by surgical extend and complexity

Type of surgery	Enucleation	Medium extensive	Extensive surgery
Number of surgeries	46 (100)	29 (100)	14 (100)
Clavien-Madadi classification(worst)			
Overall complication rate I-V^1^	18 (39.1)	21 (72.4)	9 (64.3)
I-IIIa	16 (34.8)	19 (65.5)	7 (50.0)
≥ IIIb	2 (4.35)	2 (6.9)	2 (14.3)
Clavien-Dindo Classification(worst)			
Overall complication rate I-V	18 (39.1)	21 (72.4)	9 (64.3)
I-IIIa	16 (34.8)	17 (58.6)	5 (35.7)
≥ IIIb	2 (4.35)	4 (13.8)	4 (28.6)
Mean comprehensive complication index^1^	10.28	19.73	23.71

Data presented as N(% of specific surgical extend)

The CCI is comprised of all surgeries, not the single worst

## Discussion

The goal of this study was to estimate the rate and grade of postoperative complications after surgical treatment for CHI and pediatric insulinomas in childhood and infancy based on strict predefined definitions and grading systems of complication. Recurrent disease or the inability to cure were not considered a surgical complication in this study.

Research on complications after elective pancreatic surgery in children is still very scarce; especially regarding surgery for CHI and insulinomas. In addition, international guidelines for the diagnosis and treatment of hyperinsulinism do not mention postoperative complications [1, 26]. Grounds for comparison are, therefore, very limited with only brief descriptions of postoperative complications in other cohort studies. The most viable dataset for comparison evaluated 500 pancreatectomies in CHI patients [27]. In this cohort, eight (3.25%) cases of small bowel obstruction were reported in a population of 246 focal CHI’s. The diffuse CHI population of 202 patients had four cases of small bowel obstruction, three cases of common biliary duct injury, one case of common biliary duct stricture and one intra-abdominal abscess totaling 10 (4.95%) complications. No pancreatic fistulas were reported in either group. This large study was not designed to evaluate the rate and grade of postoperative complications and therefore may carry a risk of underreporting – especially in terms of CMC grade I-IIIa complications.

Other available data include an overview of 65 pancreaticoduodenectomies for neoplasms in children [28], with a pancreatic leak rate as their most common complication, present in 14% of cases. Patients in this study had a median age of 13 years, which is significantly higher than the 6.3 months in our study population (Table 1). In addition, the more extensive pancreaticoduodenectomies and the nature of the disease in the cited study make comparison impossible. In adult populations, the rate of clinically relevant POPF(grade B and C) has been reported to occur in 14,2–17,4% of patients undergoing distal pancreatectomy and 20% of patients undergoing pancreatoduodenectomy [29, 30]. Thus highlighting the comparatively low incidence of clinically relevant POPF in this pediatric population at 3.4%. The relatively high incidence of delayed gastric emptying (20%) presented in this study should be interpreted with caution as feeding problems in CHI is a known multifactorial issue and not strictly a surgical complication. Both the presence of HI [31] and the use of anti-insulin medication [32] may contribute to the inability to return to a standard diet.

As expected, the complication rate seems to depend on the extend and complexity of the surgical procedure (Table 5*)* with 37% of enucleations resulting in at least one surgical complication, contrary to 72.4% and 64.3% in the medium extensive and the extensive surgery groups, respectively. This is further highlighted by the mean CCI values varying from 10.28 in the enucleation group to 19.73 in the medium extensive group and 23.71 in the extensive group. Consequently, the CCI values describe the cumulative burden of postoperative complications encountered in each surgical group and by proxy the severity of the complications. However, since this study is a retrospective chart review, direct comparison between surgical approaches should be viewed cautiously. Our cohort had a relatively high number of focal CHI patients (65% of all CHI subtypes) compared to other centers with approximately 50% having focal CHI [9]. We ascribe the high prevalence of focal CHI among our surgeries to a preferential referral of patients with genetically predicted focal CHI for curative surgery and increased efforts to treat diffuse CHI by complex medication and diet in our and in many other referral centers.

### Strengths and limitations

Strengths of our study include the novelty of performing a systematic evaluation of postoperative complications by use of internationally standardized classifications, the strict evaluation of journal files and the high patient number for a Scandinavian country.

We acknowledge that the retrospective design of the study inherently limits the depth of analysis, specifically concerning data collection and standardization of outcome measures. The study was intended to be a comprehensive description of a single specialist centers cumulative experience concerning the treatment and postoperative care of congenital hyperinsulinism, and should be seen as such. In addition, we adapted a definition and grading of complications used in adult patients for a pediatric cohort.

The retrospective design of this study is further limited by the quality of the medical file entries. Wrongly entered data or missing reports diminish the study’s ability to predict the rates of complications. Furthermore, the low incidence of CHI and insulinomas sets limitations on the study cohort size. A multicenter study could become a future way forward to improve the estimates of postoperative complication rates and benchmarking. Due to the international nature of our hyperinsulinism center, many patients were discharged prior to the 30-day follow-up, with a mean length of stay after surgery of 16 days, further complications may have presented themselves in the country of origin without our knowledge. Lastly, the rating and grading of the complications were done by a single author, where multiple authors separately rating complications and discussing cases would optimize the internal validity of the assessment.

## Conclusion

This retrospective study identified delayed gastric emptying, non-surgical infections, pancreatic fistula and exocrine insufficiency as the most frequent postoperative complications following surgery for CHI or insulinomas in pediatric patients. Although some complications may be unrelated to the surgical procedure itself, the overall rate of significant traditional surgery-related complications was low. Only 8% of patients experienced complications classified as Clavien-Madadi grade IIIb or higher. Future studies should be prospective and based on internationally standardized definitions and grading systems.

## Data Availability

Raw data sets are not available according to the agreement with the data protection agency(Project ID: 24/16225) and theRegional Committees on Health Research Ethics for Southern Denmark (Project ID: 24/10264). Available data is provided in the manuscript.
